# How Much Did Internet Use Promote Grain Production?—Evidence from a Survey of 1242 Farmers in 13 Provinces in China

**DOI:** 10.3390/foods11101389

**Published:** 2022-05-11

**Authors:** Yangyang Zheng, Qinqin Fan, Wei Jia

**Affiliations:** 1 Business School, Wenzhou University, Wenzhou 325035, China; zhengyangyang364@163.com; 2 School of Economics and Management, China Agricultural University, Beijing 100083, China; fanqinqina@foxmail.com; 3 Institute of Agricultural Economics and Development, Chinese Academy of Agricultural Sciences, Beijing 100081, China

**Keywords:** internet use, farmers, grain production, farmer heterogeneity

## Abstract

Increasing grain production and ensuring food security are always major issues in China, which are related to the sustainable development of the nation. The sudden outbreak of COVID-19 in 2020 has brought new challenges to global food security, which highlights the “ballast stone” and “stabilizer” role of food. China’s food security must rely on domestic production. As an important production factor, the Internet is also an important channel for farmers to obtain agricultural information, which can effectively reduce the information search cost and information asymmetry. Existing studies have mainly focused on the impact of Internet use on agricultural inputs, agricultural prices, and agricultural efficiency; there are few studies on the impact of Internet use on grain production. To fill this gap, based on the micro survey data of 1242 maize farmers in 13 provinces in China, this paper employs linear regression models and PSM methods to deeply explore the impact of Internet use on farmers’ grain production. The results show that Internet use has a significant positive impact on the grain production of farmers. Compared with the farmers who do not use the Internet, Internet use increases the maize yield of farmers by 14.25%. The heterogeneity analysis further shows that the impact of Internet use on the grain production of different farmers varies significantly: the maize yield per ha after using the Internet for farmers of younger, low education level, large-scale, and living in well-developed villages had increased by 13.65%, 15.38%, 23.94%, and 10.64%, respectively, compared with the counterfactual scenario of farmers who do not use the Internet. The results of this paper have passed the robustness test.

## 1. Introduction

“The people are the foundation of the country, and the grain is the people’s life.” Food has been the foundation of safety in the world since ancient times. China uses 7% of its arable land to feed 20% of the world’s population, creating a world miracle. China’s grain output has more than doubled from 305 million tons in 1978 to 664 million tons in 2019. Since 2003, there have been fifteen consecutive increases in grain production. In recent years, grain production has basically remained stable, but there is pressure for continued growth. China’s food self-sufficiency rate remains at about 85%, and there is still a large gap in food supply and demand, which is lower than 90% of the world’s safety standard. The outbreak of COVID-19 has highlighted the instability of supply from the world’s food market, which will definitely affect China’s food imports. The Chinese government emphasizes that “the Chinese will carry the Chinese bowl, and the Chinese bowl will carry the Chinese grain.” The Chinese government has realized that China’s food security can only be safeguarded with an increase in grain production.

Existing literature has studied many factors affecting grain production in China, such as agricultural mechanization (Xu et al., 2014; Qiao, 2017) [1,2], agricultural labor migration (Ge et al., 2018; Abate and Kuang, 2021) [3,4], fertilizer inputs (Xu et al., 2018) [5], arable land resources (Otsuka et al., 2016; Qiu, 2020) [6,7], technological progress (Jin et al., 2002) [8], agricultural policies (Liu et al., 2015; Yu and Wu, 2018) [9,10], natural disasters (Holst et al., 2013; Yang et al., 2017) [11,12], and the aging of the rural population (Liu et al., 2021) [13]. Grain prices and farmers’ income are discussed in the paper (Zhang et al., 2016; Xie and Wang, 2017) [14,15]. Many factors have different action mechanisms on grain production (Wang et al., 2013; Pan et al., 2020; Liang et al., 2021) [16,17,18]; the sown area, fertilizer inputs, and technological progress are the main factors affecting the comprehensive grain production capacity in China (Yao et al., 2001; Li et al., 2018; Yu et al., 2022) [19,20,21]. The existing literature focused more on traditional production factors, such as labor, capital, and agricultural policy, while research on how an emerging factor—Internet use—affects grain production is relatively rare.

With the popularization of the Internet, its application in agriculture has become increasingly extensive, and “Internet + agriculture” has gradually become a force in the development of agricultural modernization. As an important tool for farmers to obtain information, the Internet breaks through the time and space limitations of information dissemination. Farmers can obtain, process, and share information through the Internet. In the short term, the Internet can improve the ability of farmers to obtain information and technology, and increase agricultural production input. In the long run, it can improve the agricultural management ability of farmers (Aker and Ksoll, 2016; Rahman and Mamun, 2017) [22,23]. Whether Internet use can have an impact on food production is an important question that this paper focuses on. 

Many scholars have analyzed the extensive impact of Internet use on agriculture from different perspectives [24]. Stigler (1961) [25] discussed the role of information in reducing market transaction costs and information asymmetry. Aker (2008) [26] found that mobile phones would increase the pre-booked selling price of traders in the grain market and the number of markets searched based on Nepalese data from 2001 to 2006, thereby reducing price differences across the market. This is similar to the conclusion of Jensen (2007) [27]. However, Tadesse and Bahiigwa (2015) [28] found that the impact of mobile phones on farmers’ marketing decisions and sales prices is weak based on village-level information collected in rural Ethiopia; the main reason is that they cannot obtain the desired information through mobile phones. Similarly, Aker and Fafchamps (2013) [29] assessed the impact of mobile phones on the price differentials of agricultural products in Niger and found that mobile phone coverage reduced the spatial dispersion of producer prices for semi-perishable commodities, such as cowpea, but not for non-perishable commodities, such as millet and sorghum. Shimamoto et al. (2015) [30] further proved that whether farmers obtain information through mobile phones would affect the sales price of products based on the study of rural areas in Cambodia. Numerous studies have demonstrated that Internet use promotes agricultural production technology adoption, but the influencing mechanism is different. On the one hand, Internet use promotes farmers’ adoption of new crop varieties, organic fertilizers, and improved maize storage techniques by reducing market friction and improving bargaining power (Adegbola and Gardebroek, 2007; Aker, 2010; Zheng et al., 2022) [31,32,33]; on the other hand, it promotes the adoption of IPM (Integrated Pest Management) technologies by improving farmers’ cognitive abilities and changing agricultural production methods (Larochelle et al., 2019; Yan et al., 2019) [34,35]. Internet use ultimately affects agricultural performance, Mittal and Tripathi (2009) [36] found that the use of mobile phones can significantly improve agricultural efficiency based on the study of small farmers in India. Similar conclusions can be drawn (Aker and Ksoll, 2016; Rahman and Mamun, 2017; Zhu et al., 2019) [22,23,37]. Moreover, compared with farmers who do not use the Internet, Internet use can reduce poverty, increasing by 2643 yuan per year (Rahman and Mamun, 2017; Ma et al., 2018) [23,38]. Existing literature focuses on the impact of Internet use on agricultural prices, production, and performance. However, we find that there are few studies on the impact of Internet use on grain production, and fewer studies based on the perspective of farmers. In this paper, Internet use means that farmers use the Internet to obtain agricultural information, which includes the quantity and price of agricultural inputs (such as land, capital, labor, seeds, pesticides, and fertilizers, etc.), production technical services (such as pest control, soil testing formula, plant protection, and field management, etc.), and agricultural market and policy, etc. As of the end of 2018, the number of rural Internet users in China was 222 million, accounting for about 26.7% of the total number of Internet users. Rural Internet has developed rapidly and has huge potential. In 2019, the No. 1 Central Document clearly stated that “Internet + Agriculture” should be promoted, and the construction of digital agriculture should be strengthened. In May 2019, the Chinese government issued the “Digital Rural Development Strategy Outline”. According to the document, it is necessary to liberate and develop digital productivity, bridge the “digital divide” between urban and rural areas, and make agriculture a promising industry. This paper uses household survey data to discuss the impact of Internet use on grain production, expanding on the existing literature. The main objectives of this paper are as follows: first, we explore whether and the extent to which Internet use can affect grain production; second, we investigate the potential heterogeneous effects of Internet use on farmers’ grain production across age, education level, farm size, and the village economic development level.

The structure of this paper is as follows: first, we evaluate the existing literature, point out the deficiencies of the existing research literatures, and put forward the main objects; second, we introduce the research methods and data sources; based on the traditional farmer’s production behavior model, through theoretical derivation, the empirical research method of this paper is formed; the data used are mainly from the maize farmers, covering 116 villages in 96 counties in 13 provinces in China; third, we discuss the model results and conduct robustness analysis, and analyze the heterogeneity of the impact of Internet use on grain production from four aspects; fourth, we present research conclusions and policy implications for encouraging farmers to use the Internet to obtain agricultural information and increase grain production.

## 2. Theoretical Model

### 2.1. Theoretical Analysis

Traditional farmers’ production behavior models are mostly based on classical theoretical assumptions, which believes that the information required by farmers is complete, and the market will be cleared instantly. In fact, it is difficult for farmers to obtain complete information when making production decisions. The Internet can reduce farmers’ information costs, improve farmers’ awareness of agricultural technology, market and management, and achieve optimal allocation of resources (Aker and Ksoll, 2016; Zhou, 2016) [22,39]. The impact of the Internet on agricultural production is seen in the following four areas. First, Internet use can enable farmers to obtain more agricultural production information via different production links. For example, in the cultivation stage, by searching the weather, seeds, and fertilizer information on the Internet, farmers can choose the most suitable cultivation time, and apply chemical fertilizers scientifically; in the growing stage, farmers obtain information on agricultural pests and diseases through the Internet, so as to effectively prevent and control pests and diseases; in the harvesting stage, farmers can obtain timely and accurate weather and crop harvesting information through the Internet use, and choose the most appropriate harvesting time to avoid food loss (Adegbola and Gardebroek, 2007; Fang and Liu, 2018; Zhang et al., 2019) [31,40,41]. Second, Internet use promotes the adoption of new technologies. Internet use encourages farmers to fully understand the “risk-benefit”, avoid risks associated with new technologies, and promote the adoption of new technologies (Genius et al., 2014; Ma and Wang, 2020) [42,43]. Third, Internet use promotes farmers’ agricultural production investment and optimizes the allocation of agricultural resources (Mittal and Tripathi, 2009; Aker, 2010; González et al., 2014) [32,36,44]. The Internet is embedded in the whole process of agricultural production, which can optimize the allocation of land, capital, and labor; reduce agricultural costs; improve production efficiency; and encourage farmers to invest in agricultural production (Aker and Mbiti, 2010; Kaloxylos et al., 2013; Hou et al., 2018) [45,46,47]. Fourth, Internet use changes farmers’ traditional production concepts, exposes them to more modern management concepts, and makes them more innovative and adventurous (Camacho and Conover, 2010; Fafchamps and Minten, 2012) [48,49]. Internet use can enable farmers to acquire more new management knowledge and improve their management capabilities. Moreover, Internet use improves farmers’ environmental literacy by improving their environmental awareness, knowledge, and behaviors (Peng et al., 2019) [50]. 

Based on the above theoretical analysis, we construct a theoretical model to explore the impact of Internet use on grain yield. Farmers pursue profit maximization to make economic decisions. The farmer’s output equation *Y* is constructed:

(1) Y=A·F(K,L,S)

where *Y* is the output; *A* is the technology inputs; and *K*, *L*, *S* are the capital inputs, labor inputs, and land inputs, respectively.

Divide both sides by the land area *S*:

(2) y=Af(k,l)

where y=YS, k=KS, l=LS.

In Equation (2), it is assumed that farmers are homogeneous. In order to overcome the defects brought by the homogeneity assumption, in the empirical analysis, this paper introduces the characteristics of household head, household, village, and regions to control the heterogeneity, and introduces the core variable Internet use. Equation (2) is converted into Equation (3):

(3) y=Af(k,l,γ,region,internet)

where γ is the set of control variables, including household head characteristics, household characteristics, and village characteristics; region is the regional characteristics; and internet is the use of the Internet.

The specific form is as follows:

(4) $${\mathrm{Maize}}_{i}=\beta_{0}+\beta_{1}internet_{i}+\varphi z_{i}+\delta region_{i}+\varepsilon_{i}$$

where ${\mathrm{Maize}}_{i}$ is the grain production of the farmer; $internet_{i}$ is the core variable of the farmer’s Internet use; $\beta_{1}$ is the coefficient of Internet use; $z_{i}$ is the control variable that affects the farmer’s grain production; φ is the coefficient of the control variable; $region_{i}$ is the regional dummy variable; and *δ* is the coefficient of regional dummy variable.

### 2.2. Propensity Score Matching

Since the initial conditions of the farmers who use the Internet and those who do not use the Internet are not exactly the same, such as age, education, and risk preference, etc., there may be selection bias if we regress directly. The propensity score matching method (PSM) constructs a “counterfactual” framework by finding a counterfactual control group similar to the treatment group to eliminate the sample selection bias to the greatest extent. (Rosenbaum and Rubin, 1983; Caliendo and Kopeinig, 2008; Abadie and Imbens, 2016) [51,52,53]. Specific steps are as follows:

First, we use the Logit model to estimate the probability of farmers using the Internet and estimate the propensity score, as shown in Equation (5):

(5) $$P\left(X_{i}\right)=F\left({\mathrm{Internet}}_{i}=1|X_{i}\right)=\frac{1}{1+e^{-X_{i}B}}$$

where $P\left(X_{i}\right)$ is the probability of farmer i using the Internet, $X_{i}$ is the influencing factor of farmer Internet use, and ***B*** is the coefficient of the influencing factor. 

Second, we use nearest neighbor matching, radius matching, kernel matching, and local linear regression matching to obtain the Treatment Group and Control Group, thereby eliminating the problem of self-selection (Ji et al., 2019) [54].

Nearest neighbor matching. According to the propensity score value estimated by the Logit model, we find the control group samples that are closest to the propensity score value in the treatment group samples. Suppose C(i) is the sample set that matches the *i*-th sample in the treatment group, and $p_{i}$ is the propensity score value, the specific equation is as follows:

(6) $$C\left(i\right)=\underset{j}{\min}\left|p_{i}-p_{j}\right|$$

Radius matching. The closest matching in reality may also be far from the actual, thus losing comparability; therefore, the radius matching method is adopted. That is, we restrict the absolute distance of the propensity score value, assuming $\left|p_{i}-p_{j}\right|\leq 0.25\hat{\sigma }$. $\hat{\sigma }$ is the sample standard deviation of the propensity score.

Kernel matching. Essentially, both nearest neighbor matching and radius matching belong to nearest neighbor matching; that is, a simple arithmetic average is performed on the nearest neighbor matching samples. Kernel matching gives different weights according to individual distances. Generally speaking, the closer the distance is, the greater the weight, and the value is 0 beyond a certain range (Nadaraya, 1964; Watson, 1964) [55,56]. 

We consider building a nonparametric univariate regression model:

(7) $$y_{i}=m\left(x_{i}\right)+\varepsilon_{i}\;\varepsilon_{i}\sim iid\left(0,\sigma_{\varepsilon }^{2}\right)$$

where m(.) is an unknown function and $\varepsilon_{i}$ is a random error term.

Supposing a certain value of *x*, such as $x_{0}$, $y_{j}$ is the observation value corresponding to a nearest neighbor $x_{0}$, and $Y_{0}$ is the “local weighted average estimator”; that is, the weighted average of the observation value corresponding to a nearest neighbor $x_{0}$.

(8) $$Y_{0}=\sum_{i=1}^{n}w\left(i,j\right)y_{i}$$

where w(i,j) is the weight, the weight expression of the kernel equation is as follows:

(9) $$w\left(i,j\right)=\frac{K\left[\left(x_{j}-x_{i}\right)h\right]}{\sum_{i=1}^{n}K\left[\left(x_{j}-x_{i}\right)h\right]}$$

where *h* is the broadband; K(.) is the kernel equation; and $x_{j}$ is the point near $x_{i}$.

Local linear regression matching. Kernel matching is essentially a “local constant estimator”. Local linear regression matching can not only solve the “boundary problem” well, but also is more efficient and applicable to more data types than kernel matching (Fan, 1992) [57]. The specific equation is as follows:

(10) $$\underset{\left|a_{0},b_{0}\right|}{\min}\sum_{i=1}^{n}K\left[x_{i}-x_{0}/h\right]{\left[y_{i}-a_{0}-b_{0}\left(x_{i}-x_{0}\right)\right]}^{2}$$

where *h* is the broadband; K(.) is the kernel function; $y_{j}$ is the observation value corresponding to a nearest neighbor $x_{0}$, assuming that m(x) is a linear function in a nearest neighbor $x_{0}$, and then $m\left(x\right)=a_{0}-b_{0}\left(x_{i}-x_{0}\right)$; $a_{0}$ is the constant term; and $b_{0}$ is the coefficient of $\left(x_{i}-x_{0}\right)$.

Third, according to the matching samples obtained above, we compare the average difference between the grain production of farmers in the treatment group and the control group. The “Average Treatment Effect on the Treated” (ATT) is defined as:

(11) $$ATT=E\left[\left(Y_{1}-Y_{0}\right)|D=1\right]=E\left\{E\left[\left(Y_{1}-Y_{0}\right)|D=1\right],P\left(X\right)\right\}$$

where *D* is treatment variable of 0 and 1; in other words, *D* = 1 represents the treatment group, *D* = 0 is the control group, *P*(*X*) is the propensity score value, and *Y*_1_ and *Y*_0_ are the estimated results on maize yield of farmers using the Internet and not using the Internet.

## 3. Data and Variable Selection

### 3.1. Data

The data for this study comes from household surveys conducted from January to February 2019 by the National Agricultural and Rural Development Research Institute of China Agricultural University. The investigators are undergraduate, masters, and doctoral students among various majors of China Agricultural University. The questionnaires mainly focus on the main maize-producing areas in China. Firstly, the research team determines the number of samples in different provinces and cities based on maize production. We selected 13 major maize-producing provinces, namely, Inner Mongolia, Jilin, Sichuan, Anhui, Shandong, Jiangsu, Jiangxi, Hebei, Henan, Hubei, Hunan, Liaoning, and Heilongjiang. Secondly, we sampled according to the surveyed counties (cities) corresponding to the corresponding household registration students. The students conducted surveys in the township where the household registration is located, and selected about 1–2 villages, about 15 households. Ultimately, 1242 farmer questionnaires were obtained from 13 provinces (autonomous regions), 96 counties (districts), and 116 villages. Thirdly, a number of researchers were responsible for sending, receiving questionnaires, and answering questions encountered during the investigation. We organized special training activities before the investigation to explain the questionnaire. On the whole, the sample covers the eastern, central, western, and northeastern regions of China. At the same time, the major grain-producing provinces are also fully considered, so the sample is relatively representative (see Table 1).

### 3.2. Variable Selection

We selected household grain production as dependent variable, which is expressed as a household’s maize yield per ha. We divided the total farm household maize output in 2018 by the household’s maize sown area to get the household’s maize yield per ha.

Independent variables include core independent variables and control variables. The core independent variable is Internet use. We considered that even if farmers have mobile phones or strong Internet skills, they may not obtain agricultural information through the Internet. We draw on the research of Ma et al. (2021) and Nie et al. (2021) [58,59], and Internet use is represented by a dummy variable; that is, whether farmers use the Internet to obtain agricultural information or not. If farmers use the Internet to obtain agricultural information, the value is 1; otherwise, the value is 0.

Control variables. According to existing research, farmers’ grain production is affected by many factors. In addition to the Internet use that this paper focuses on, it is also affected by other variables. Referring to Yang et al. (2019) and Ma et al. (2020) [60,61], the household head characteristics include age, education, health, training, and risk preference. Referring to Boz (2016) and Hoffmann and Kelly (2018) [62,63], household characteristics include the proportion of non-agricultural income, farm size, number of plots, and subsidies. Referring to Janssen and Bert (2006) and Wang et al. (2011) [64,65], production inputs include seed costs, pesticide costs, fertilizer costs, irrigation costs, machinery costs, and labor input. Referring to Tatlıdil et al. (2009) [66], village characteristics include Whether it is a poor village and the economic development level of village. The statistical description of the variables is shown in Table 2. 

The statistical characteristics of the sample of farmers are as follows. The average age of the heads of household who use the Internet is 48.55, which is lower than the age of the farmers who do not use the Internet, showing that the farmers who use the Internet are younger. The average education level of the farmers who use the Internet is 3.13, which is higher than the 2.70 of farmers who do not use the Internet. That is to say, the education level of the farmers who use the Internet is high, and they are basically above junior high school. The risk preference of farmers who use the Internet is higher than that of farmers who do not use the Internet. The farm size of farmers who use the Internet is 1.95 ha, which is larger than the 1.27 ha of farmers who do not use the Internet. Namely, farmers with large-scale farms are more inclined to use the Internet in the production process. The number of plots owned by farmers using the Internet is 4.28, lower than the 5.42 plots owned by farmers not using the Internet. The agricultural subsidy received by farmers that use the Internet is 2365.409 RMB, which is higher than the 1901.31 RMB for non-Internet farmers. The pesticides inputs per ha for Internet-using farmers was 450.32 RMB, which was lower than 563.50 RMB for non-Internet farmers. The cost of fertilizer per ha for farmers who use the Internet is 2849.54 RMB, which is 2581.76 RMB higher than the cost of fertilizer for farmers who do not use the Internet. The cost of machinery per ha for farmers who use the Internet is 1556.39 RMB, which is higher than the 1353.98 RMB for farmers who do not use the Internet. The economic development level of the villages where Internet users live is higher than the villages where the non-Internet users live. 

**Figure 1:**
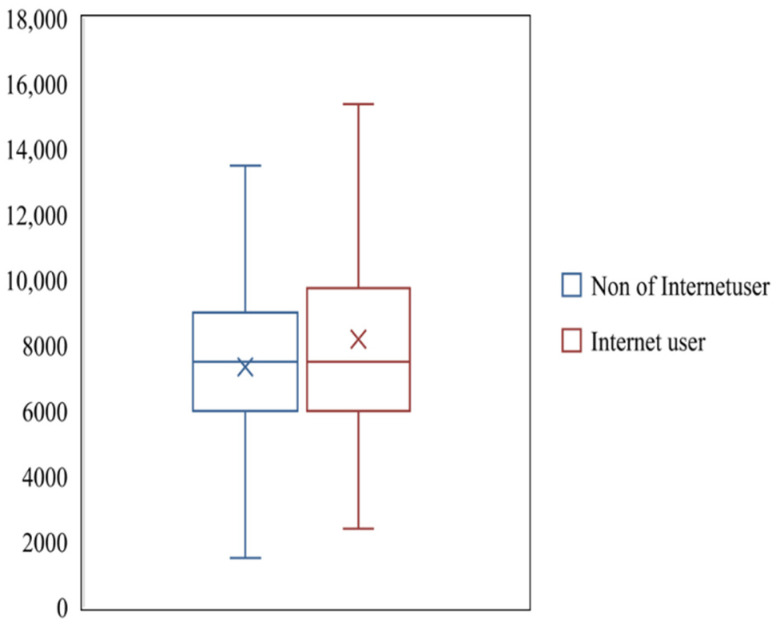
The difference in food production between Internet-using farmers and non-Internet-using farmers.

**Figure 2:**
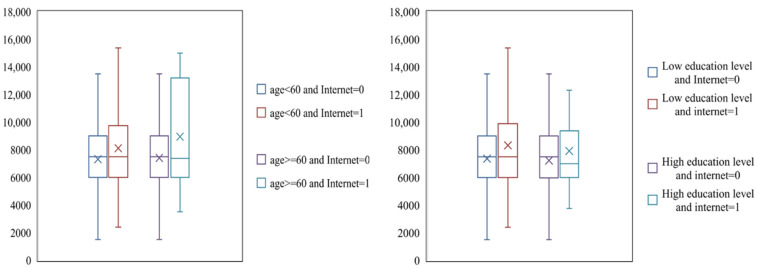
Grain production of Internet-using farmers and non-Internet-using farmers under different age and education levels.

**Figure 3:**
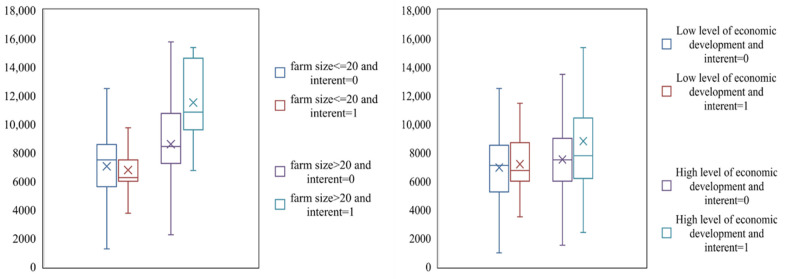
Grain production of Internet-using farmers and non-Internet-using farmers under different farm size and economic development levels.

From Figure 1, Figure 2 and Figure 3, it can be seen that the average maize yield of farmers who use the Internet is 8192.27 kg per ha, which is higher than the 7346.17 kg per ha of farmers who do not use the Internet. For farmers below 60, the average maize yield of farmers using the Internet was 8108.03 kg per ha, which was higher than that of farmers not using the Internet, 7316.03 kg per ha. For farmers aged 60 or more, the average maize yield of farmers who use the Internet is 8946.24 kg per ha, which is higher than that of farmers who do not use the Internet, 7412.16 kg per ha. For farmers with a low education level, the average maize yield of farmers using the Internet is 8200.73 kg per ha, which is higher than 7367.47 kg per ha for farmers who do not use the Internet. For farmers with high education level, the average grain output of farmers who use the Internet is 7921.32 kg per ha, higher than the 7230.31 kg per ha of farmers who do not use the Internet. For farmers of farm size is less than or equal to 1.33 hectares (1 hectare = 15 mu), the average maize yield of farmers using the Internet is 6792.05 kg per ha, which is lower than that of farmers not using the Internet, 7048.59 kg per ha. For farmers of farm size is more than 1.33 hectares, the average maize yield of farmers using the Internet is 11,514.85 kg per ha, which is much higher than that of farmers not using the Internet, 8592.72 kg per ha. For farmers living in undeveloped village, the average maize yield of farmers who use the Internet is 7196.09 kg per ha, which is higher than that of farmers who do not use the Internet, 64.9.18 kg per ha. For farmers living in well-developed village, the average maize yield of farmers using the Internet is 8807.8 kg per ha, much higher than the 7521.87 kg per ha of farmers who do not use the Internet.

## 4. The Impact of Internet Use on Grain Production

### 4.1. Regression Results of Linear Regression Model

In this paper, the variance inflation factor (VIF) was used to test the multicollinearity, and the value is 1.36 (<10); it indicates that there is no multicollinearity. The robust standard error method was used in the regression to overcome the heteroscedasticity problem (Wooldridge, 2015) [67]. From the overall regression results in Table 3, the fitting degree of the model is relatively good. Internet use has a significant impact on grain production, which indicates that compared with the farmers who do not use the Internet, Internet use increases the maize yield of farmers by 1066 kg per ha, by factors of 14.25%. From the results of Models 2–5, the impact of Internet use on farmers’ grain production is different in coefficient. The coefficient of education level variable is positive and statistically significant at the 1% level. This implies that the increase of one unit of education level can increase farmers’ yield by 25.28 kg per ha. The coefficient of risk preference variable is negative and statistically significant at the 1% level; that is, with the increase in risk level, farmers’ maize yield per ha decreases. Indeed, when risk preference increases by one level, farmers’ maize yield decreases by 653.6 kg per ha. When the proportion of non-agricultural income increases by one unit, farmers’ maize yield decreases by 925 kg per ha. A high share of non-agricultural income means that the household does not rely on agricultural income as their main income source, resulting in less attention paid to agriculture and production input, leading to a decline in maize yield (Babatunde et al., 2010) [68]. The coefficient of farm size is positive and statistically significant at the 1% level. From the current farm size of China’s grain, grain planting still does not reach the scale economy. With the increase in farmers’ farm size, the grain output still shows an increasing trend. This implies that as the farm size increases by 1%, farmers’ maize yield increases by 806.1 kg per ha (Samberg et al., 2016) [69]. The number of plots has a negative impact on the maize yield and has passed the significance test. This suggests that when the plot increase one unit, the maize yield will decrease by 2.519 kg per ha. The coefficient of seed fee is negative and statistically significant at the 1% level. This implies that with an increase in seed cost by 1%, farmers’ maize yield decreases by 46.01 kg per ha, which may be due to the fact that a moderate amount of maize seeds is required for a certain amount of land, and too much seed inputs may reduce maize yield. The coefficient of pesticide fee is positive and statistically significant at the 1% level. The results show that the amount of pesticide input increased by 1%, and the maize yield of farmers increased by 9.794 kg per ha, which also reflects that agricultural production still depends on pesticides to a certain extent. The coefficient of fertilizer fee is positive and statistically significant at the 5% level. This suggests that the amount of fertilizer input increased by 1%, and the maize yield of farmers increased by 20.46 kg per ha, indicating that fertilizer is the “food” of grain (Jaja and Barber, 2017) [70]. The coefficient of irrigation input variable is positive and statistically significant at the 1% level, suggesting that when irrigation input increases by 1%, farmers’ maize yields increase by 102.1 kg per ha, which indicates the importance of water security for grain production (Gordon et al., 2010; Davis et al., 2018) [71,72]. The coefficient of machinery cost variable is negative and statistically significant at the 1% level, implying that when machinery cost increases by 1%, farmers’ maize yields decrease by 132.9 kg per ha. This may be because the increase in machinery cost will reduce other productive inputs, thus reducing the maize yield per ha. Compared with non-poor villages, the maize yield per ha of farmers in poor villages decreased by 777.6 kg, which may be due to the credit constraints of farmers, so farmers have limited investment in agricultural production in poor villages. Our survey data also show that compared with poor villages, the input of pesticides and fertilizers in non-poor villages increased by 39.41% and 20.27% per ha, respectively. The economic development level of the villages where the farmers live has a negative impact on the farmers’ grain production, through the significant test. The results show that when the economic development level reduces by one level, the maize yield reduces by 291 kg per ha.

### 4.2. Propensity Score Matching Results

There is no endogenous problem of mutual causation between Internet use and grain production; in this paper, the generation of endogeneity of independent variables is more a matter of sample self-selection. This paper uses the Propensity Score Matching (PSM) method to solve the self-selection problem. The results of PSM are shown in Table 4. Since PSM has many matching methods, to make the results more robust, neighbor matching, kernel matching, local linear matching, and radius matching methods were used to obtain the ATT of farmers using Internet and not using Internet (treatment group and control group). The result shows that the coefficient of ATT is positive and statistically significant at the 1% level; the average value of the ATT for the four matching methods is 1041.53 kg per ha. Compared with the counterfactual scenario of farmers who do not use Internet, the maize yield per ha of farmer using Internet had increased by 13.92%. This result is basically consistent with the OLS method, which confirms the robustness of the research results.

The balanced test is conducted to examine whether the matching result is better balanced data. After matching, the bias of most variables becomes smaller and most T tests are not significant. The original hypothesis that there is no significant difference between the treatment group and the control group is not rejected, which suggests that using PSM is appropriate and we accept no systematic differences between the treatment and control groups (see Table 5). Compared with the results before matching, the standardized deviations of most variables have been greatly reduced, and only the deviation of the variable seed fee has increased, which does not affect the robustness of the PSM results. Furthermore, from the common value ranges of propensity score, of the total 1242 observations, two observations in the control group are no longer in the common value ranges, and seven observations in the treatment group are not in the same range. This shows that most of the observed values are in the common ranges and propensity score matching only loses a few samples.

The PSM method only controls the influence of observable variables on the results, and the unobservable variables may lead to hidden bias. In other words, Internet use may be affected by unobservable variables. Rosenbaum (2002) [73] introduced bound analysis to evaluate the degree of influence of unobservable variables on PSM results; Γ represents the degree of hidden bias, Γ = 1 represents the benchmark scenario without hidden bias, and a bigger Γ means the presence of bigger hidden bias (Dillon, 2011) [74]. Considering that the maize yield of our sample farmers is 7481.74 kg per ha in the paper, the sample of farmers less than 11,222.61 kg per ha (7481.74 × 1.5) account for 90% of the total sample of farmers. Therefore, we set the value range of Γ ∈[1,1.5]. For each value of Γ, we report the upper and lower bound significance levels, which indicate that the PSM results are still reliable even at a given degree of hidden bias. Specifically, when Γ is less than or equal to 1.2, the result is statistically significant at the 1% level; when Γ is in the 1.2–1.4 interval, the result is statistically significant at the 5% level; and when Γ is 1.45 and 1.5, the result is statistically significant at the 1% level. It shows that the PSM results are still reliable even if the hidden bias increases. The results of the bound analysis are shown in Table 6.

Additionally, compared with the linear regression results, the PSM results obtained by using different matching methods are consistent, and the difference remains within 5%. This further proves the robustness and reliability of the conclusions.

## 5. Heterogeneity Analysis

Generally, the above research analyzes the impact of Internet use on farmers’ maize yield; that is to say, it compares and analyzes the differences of maize yield per ha between Internet users and non-Internet users, but does not consider the heterogeneity of farmers. The impact of Internet use on the farmers of different ages, education levels, farm size, and village economic development level can be different. This paper analyzes from the following four aspects and the results of the heterogeneity analysis are shown in Table 7 (the $\bar{ATT}$ is the average ATT value of four matching methods):

Considering the aging of the rural population in China, this paper regards the Chinese male retirement age of 60 as the dividing line, and the farmers are divided into two groups: those who are 60 years old and above, and those who are below 60 years old. The results show that the ATT value of farmers below 60 is positive and statistically significant, suggesting that Internet use has a significant impact on the grain production of farmers below 60. The maize yield per ha of farmer using Internet had increased by 1021.18 kg (13.65%) compared with the counterfactual scenario of farmers who do not use Internet. A possible reason for these results is that, compared to the farmers over 60, those below 60 have a stronger ability to obtain and process information, which can significantly improve farmers management capability (Zhou et al., 2020) [75]. Internet use has no significant impact on the grain production of farmers over 60. A possible reason is that older farmers seldom use smart phones or computers to obtain information through the Internet, and they are unlikely to use these technologies in agricultural production.

Considering that the education level in rural areas is generally low, the farmers with an education level of junior high school and below are classified as low education-level farmers, and those with education levels of senior high school and above are classified as high education-level farmers. The results show that the maize yield per ha of farmer using Internet at the low education level had increased by 1150.77 kg (15.38%) compared with the counterfactual scenario of farmers who do not use the Internet, and the maize yield per ha of farmers using the Internet at the high education level had increased by 1012.45 kg (13.53%) compared with the counterfactual scenario of farmers who do not use the Internet. A possible reason is that farmers with low education levels have a lower information acquisition capability. Internet use can significantly improve their ability to receive and process information (Mango et al., 2013) [76].

Referring to the research of Xu et al. (2011) [77], and according to the sample distribution, this paper classifies the farmers with a farm size of 1.33 hectares or less as small-scale farmers and those with a farm size of 1.33 hectares or more as large-scale farmers. The data shows that the ATT of large-scale farmers is positive and statistically significant. The maize yield per ha of large-scale farmers using Internet had increased by 1790.94 kg (23.94%) compared with the counterfactual scenario of farmers who do not use Internet. For large-scale farmers, agriculture is their major income source. To maximize profits and optimize the allocation of agricultural production inputs, they are better able to obtain agricultural information through the Internet. Internet use has a greater effect on large-scale farmers’ grain production (Herrero, 2017) [78]. 

The economic development level in the average and above are classified as well-developed village and those below the average are classified as undeveloped village. The ATT value of farmers living in well-developed villages is positive and statistically significant; that is, the impact of Internet use on the grain production of farmers living in well-developed villages is more obvious. The maize yield per ha of farmers using Internet living in well-developed village had increased by 795.91 kg (10.64%) compared with the counterfactual scenario of farmers who do not use the Internet. A possible reason is that the well-developed village have better agricultural production conditions, and farmers may be able to obtain effective agricultural production information through the Internet to improve production input and maize yield (Bosiu and Vilakazi, 2020) [79]. For farmers living in undeveloped villages, whether they use the Internet or not has no obvious impact on the maize yield, which indirectly reflects the “Matthew effect” of Internet use on farmers’ maize yield. 

Heterogeneity analysis further confirms the promotion effect of Internet use on farmers’ grain production. From the results of farmers’ heterogeneity, Internet use has significant differences in grain production among farmers of different ages, education levels, farm size, and the village economic development level, especially on large-scale farmers’ grain production. However, the matching results of the different methods are different.

## 6. Research Conclusions and Policy Implications

This paper used the data of 1242 maize farmers collected by the National Agricultural and Rural Development Research Institute of China Agricultural University. The results show that Internet use can significantly improve farmers’ maize yield per ha. Compared with farmers without Internet use, Internet use can increase farmers’ maize yield by 1066 kg per ha, or 14.25%. The PSM results show that the maize yield per ha of farmer using Internet had increased by 1041.53 kg per ha, or 13.92%, compared with the counterfactual scenario of farmers who do not use the Internet; it confirms that the results are robust. Taking into account the heterogeneity of farmers, the impact of Internet use on grain production is obvious. The maize yield per ha of a younger farmer using the Internet, low education level, large-scale, and living in a well-developed village had increased by 13.65%,15.38%, 23.94%, and 10.64% compared with the counterfactual scenario of farmers who do not use Internet, respectively. This study also shows that improving farmers’ education level, expanding farmers’ farm size, and increasing fertilizer input may be important means to further promote farmers’ maize yields per ha. 

There are also deficiencies in this study. First, the maize farmers’ samples obtained in this paper were not randomly selected. According to the maize planting condition in 13 provinces, students from corresponding regions are selected to participate in the survey, which may lead to a slight difference between the sample distribution and the actual situation. Most of the samples are obtained based on the social relations of investigators rather than strict sampling standards for random sampling, which may have some influence on the research results. Second, the use of PSM to assess the impact of Internet use on grain yield is based on observable variables, without considering the unobservable variables. Certainly, judging from the situation of observable and unobservable variables in this paper, the unobservable variables account for a relatively small proportion. Third, the design of the variable “Internet use” may be imperfect in this paper. Internet use refers only to whether or not the Internet is used; the amount or difference of agricultural information obtained is not considered, which may have a certain degree of influence on the research results. The research of Ma et al. (2020) and Nie et al. (2020) [58,59] shows that the main way of Internet use is that farmers use computers and mobile phones. A possible future research direction is how the different ways and content of Internet information acquisition influence a farmer’s grain production.

This paper to discuss the impact of Internet use on grain production, and the conclusion is that Internet use has a significant positive impact on food production. It sets a solid theoretical and factual basis for China to implement the “Internet plus agriculture” action plan. The paper puts forward three suggestions: First, the government should encourage and guide farmers to use the Internet to obtain agricultural information. Our paper shows that Internet use can improve grain production, but only 16% of farmers in the sample used the Internet to obtain agricultural information. The government should strengthen the training of farmers on the Internet use, so that farmers can form the awareness of obtaining agricultural information through the Internet, and improve the ability to use the Internet. Second, policy formulation and implementation should fully consider the differences among farmers. Internet use has a greater effect on the grain production of farmers with low education levels, because farmers with low education levels have poor information acquisition and processing ability. Therefore, we should focus on understanding the barriers to Internet use among low-education farmers, and implement precision training for them. Third, grain production should further expand farm size. Expanding farm size can significantly increase the grain output per ha, and the Internet has a greater effect on increasing grain production among large-scale farmers. The government should establish a land transfer service platform, accelerate the promotion of land transfer, and promote contiguous agricultural operation.

## Figures and Tables

**Table 1:** Sample distribution unit: number.

Province	Sample Size	Percentage (%)	Cumulative Percentage (%)	Province	Sample Size	Percentage (%)	Cumulative Percentage (%)
Inner Mongolia	176	14.17	14.17	Henan	199	16.02	85.99
Jilin	131	10.55	24.72	Hubei	39	3.14	89.13
Sichuan	125	10.06	34.78	Hunan	15	1.21	90.34
Anhui	11	0.89	35.67	Gansu	16	1.29	91.63
Shandong	257	20.69	56.36	Liaoning	52	4.19	95.81
Jiangsu	15	1.21	57.57	Heilongjiang	52	4.19	100.00
Hebei	154	12.40	69.97	Total	1242	100	100

**Table 2:** Descriptive statistics of the variables.

Variable	Variable Description	All	Internet User	Not Internet User	Difference
Maize yield per ha	kg	7481.74	8192.27	7346.17	846.107 ***
Age	Age of household head, in years	52.79	48.55	53.60	−5.043 ***
Education	Illiteracy = 1; elementary school = 2; junior high school (secondary vocational) = 3; high school (secondary vocational) = 4; junior college (higher vocational) = 5; college or higher = 6	2.76	3.13	2.70	0.430 ***
Health	Good = 1; Normal = 2; Poor = 3; No labor capacity = 4	1.42	1.37	1.43	−0.065
Train	1 if smallholder farmers receive training, 0 otherwise	0.19	0.22	0.18	0.038
Risk preference	Risk conservative type = 1; risk neutral type = 2; risk preference type = 3	1.40	1.51	1.38	0.130 ***
Proportion of non-agricultural income	The proportion of household non-agricultural income in total household income	0.60	0.63	0.59	0.034
Farm size	Logarithm of farm size (unit: ha)	0.61	0.71	0.59	0.119 ***
Number of plots	(unit: plots)	5.23	4.28	5.42	−1.134
Subsidy	Logarithm of subsidies in total (it includes agricultural machinery subsidies, subsidies for large grain farmers, production technology subsidies, agricultural insurance premium subsidies, loan discounts, etc.): (unit: RMB)	6.29	6.68	6.21	0.469 ***
Seed fee	(unit: RMB)	6.67	6.71	6.66	0.048
Pesticide fee	(unit: RMB)	5.77	5.47	5.83	−0.361 ***
Fertilizer fee	The cost of chemical fertilizer and organic fertilizer (unit: RMB)	7.68	7.82	7.65	0.167 ***
Irrigation cost	The cost of electricity and irrigation (unit: RMB)	4.79	4.55	4.84	−0.295
Machinery cost	The cost of machinery operation (unit: RMB)	6.38	6.95	6.27	0.677 ***
Invest time	(unit: day)	2.03	2.06	2.03	0.026
Whether or it is a poor village	1 if it is a poor village, 0 otherwise	0.26	0.29	0.25	0.043
Economic development level	Good = 1; better = 2; general = 3; poor = 4; very poor = 5	3.29	3.47	3.26	0.215 ***

Note: *** is significant at the 1% levels.

**Table 3:** The linear regression results of the impact of Internet use on grain production.

Variables	Maize Yield per ha				
	Model 1	Model 2	Model 3	Model 4	Model 5
Internet use	1066 ***	958.5 ***	795.6 ***	944.8 ***	959.8 ***
	(165.9)	(170.3)	(174.3)	(200.0)	(199.5)
Age	7.714	10.81	6.815	3.266	
	(6.959)	(6.992)	(7.056)	(7.400)	
Education	260.3 ***	296.1 ***	270.8 ***	265.1 ***	
	(80.00)	(80.66)	(81.51)	(84.15)	
Health	25.28	−48.96	−26.91	−106.6	
	(99.69)	(100.2)	(102.5)	(105.4)	
Train	−72.71	135.4	118.9	149.1	
	(187.4)	(185.8)	(177.6)	(190.5)	
Risk preference	−653.6 ***	−667.1 ***	−639.2 ***	−688.7 ***	
	(116.9)	(119.3)	(121.0)	(123.9)	
Non-agricultural income proportion	−925.0 ***	−874.8 ***	−1077 ***		
	(220.4)	(224.3)	(230.9)		
Farm size	806.1 ***	874.7 ***	966.6 ***		
	(164.5)	(166.4)	(164.6)		
Number of plots	−46.01 ***	−47.49 ***	−47.55 ***		
	(13.49)	(13.77)	(12.83)		
Subsidy	28.61	26.48	26.03		
	(41.04)	(40.44)	(40.57)		
Seed fee	−180.8	−156.7			
	(137.9)	(140.9)			
Pesticide fee	151.9 ***	155.6 ***			
	(46.75)	(47.67)			
Fertilizer fee	252.2 **	314.6 ***			
	(103.3)	(106.7)			
Irrigation cost	102.1 ***	114.3 ***			
	(26.36)	(26.67)			
Machinery cost	−132.9 ***	−150.4 ***			
	(39.19)	(39.65)			
Invest time	−94.92	−107.0			
	(83.40)	(82.12)			
Whether or not it is a poor village	−777.6 ***				
	(175.2)				
Economic development level	−291.0 ***				
	(90.44)				
Eastern reference group					
Middle region	−1288 ***	−1410 ***	−1450 ***	−1558 ***	−1525 ***
	(162.4)	(164.2)	(164.7)	(163.8)	(160.0)
Western region	418.6 *	224.3	310.3	699.0 ***	546.5 **
	(227.3)	(234.7)	(221.0)	(228.5)	(233.4)
Northeast region	638.6 ***	524.6 ***	320.0 *	1195 ***	1032 ***
	(191.7)	(196.6)	(190.6)	(174.6)	(169.6)
Constant	7348 ***	5495 ***	7467 ***	7442 ***	7317 ***
	(1218)	(1134)	(588.0)	(562.9)	(114.2)
Observations	1242	1242	1242	1242	1242
Pseudo R^2^	0.285	0.259	0.223	0.145	0.115

Note: Robust standard errors are shown in brackets; ***, **, and * are significant at the 1%, 5%, and 10% levels, respectively.

**Table 4:** PSM results of the impact of Internet use on grain production.

Matching Method	Treatment Group	Control Group	ATT	Standard Error	T Value
Nearest neighbor matching	8248.11	7170.05	1078.07 ***	266.29	4.05
Kernel matching	8248.11	7221.24	1026.88 ***	244.51	4.2
Local linear regression matching	8248.11	7200.57	1047.54 ***	333.46	3.14
Radius matching	8248.11	7234.48	1013.63 ***	244.62	4.14

Note: Standard errors are shown in brackets; *** is significant at the 1% levels. The kernel matching broadband selects the default value of 0.06.

**Table 5:** Balance test of the PSM results.

Variable	Unmatched/ Matched	Treated	Control	Bias (%)	Reduct |Bias|	*T* Test	*p* > t
Age	U	48.55	53.60 ***	−48.8		−5.94	0.000
	M	48.85	47.98	8.4	82.8	0.84	0.404
Education	U	3.13	2.70 ***	46.8		6.09	0.000
	M	3.07	3.15	−8.6	81.6	−0.82	0.411
Health	U	1.37	1.43	−10.7		−1.33	0.185
	M	1.37	1.32	8.6	20.1	0.88	0.380
Train	U	0.22	0.18	9.5		1.25	0.210
	M	0.19	0.18	4.3	54.3	0.44	0.662
Risk preference	U	1.51	1.38 ***	20.4		2.74	0.006
	M	1.51	1.58	−11.6	42.9	−1.05	0.296
Non-agricultural income Proportion	U	0.63	0.59	9.4		1.23	0.217
	M	0.62	0.62	−1.8	80.7	−0.18	0.857
Farm size	U	0.71	0.59 ***	18.1		2.59	0.010
	M	0.71	0.71	0.4	98	0.03	0.974
Number of plots	U	4.28	5.42	−15.7		−1.62	0.106
	M	4.35	4.48	−1.8	88.5	−0.32	0.746
subsidy	U	6.68	6.21 ***	25.6		3.13	0.002
	M	6.69	6.74	−2.7	89.4	−0.3	0.764
Seed fee	U	6.71	6.66	9.6		1.08	0.282
	M	6.72	6.80	−16.8	−74.9	−1.57	0.117
Pesticide fee	U	5.47	5.83 ***	−22.1		−3.2	0.001
	M	5.61	5.69	−4.9	77.9	−0.47	0.638
Fertilizer fee	U	7.82	7.65 ***	22.7		2.9	0.004
	M	7.81	7.81	−0.5	97.6	−0.06	0.955
Irrigation fee	U	4.55	4.84	−10		−1.33	0.184
	M	4.65	4.64	0.3	96.5	0.03	0.973
Machinery cost	U	6.95	6.27 ***	33		3.71	0.000
	M	6.92	6.90	1.1	96.6	0.14	0.890
Invest time	U	2.06	2.03	3.1		0.36	0.721
	M	2.03	2.04	−1.2	60.3	−0.13	0.898
Whether or it is a poor village	U	0.29	0.25	9.7		1.28	0.201
	M	0.30	0.28	5.3	45.7	0.5	0.614
Economic development level	U	3.47	3.26	24.7		3.24	0.001
	M	3.48	3.39	10.8	56.1	1.09	0.275

Note: *** is significant at the 1% levels.

**Table 6:** Rosenbaum bound analysis for grain production.

Γ	Sig+	Sig−
1.0.	0.000142	0.000142
1.05	0.000421	0.000043
1.10	0.001101	0.000013
1.15	0.002564	3.70 × 10^−6^
1.20	0.005408	1.10 × 10^−6^
1.25	0.010455	2.90 × 10^−7^
1.30	0.018722	7.90 × 10^−8^
1.35	0.031332	2.10 × 10^−8^
1.40	0.049385	5.60 × 10^−9^
1.45	0.073802	1.40 × 10^−9^
1.50	0.105186	3.70 × 10^−10^

Note: Gamma is log odds of differential assignment due to unobserved factors. Sig+ and Sig− are upper and lower bound significance level, respectively.

**Table 7:** Analysis of heterogeneity based on family characteristics.

		Age		Education		Farm Size		Economic Development Level	
		<60	≥60	Low Education Level	High Education Level	Small-Scale Farmer	Large-Scale Farmer	Undeveloped Village	Well-Developed Village
Matching method	Nearest neighbor matching	1036.81 ***	229.90	1281.90 ***	1082.65 **	71.25	2022.84 ***	198.61	728.10 *
		(277.95)	(1121.81)	(327.52)	(482.68)	(237.49)	(611.46)	(382.79)	(425.69)
	Kernel matching	1018.57 ***	318.11	1121.03 ***	974.01 **	155.61	1709.57 ***	255.49	773.50**
		(253.47)	(1086.73)	(298.79)	(475.66)	(205.93)	(604.22)	(367.49)	(383.23)
	Local linear regression matching	1001.96 ***	181.85	1080.19 ***	1017.02 *	109.95	1718.18 **	234.33	903.06*
		(331.61)	(1399.20)	(421.08)	(580.43)	(324.00)	(734.57)	(556.73)	(479.61)
	Radius matching	1027.39 ***	320.03	1119.97 ***	976.12 **	140.91	1713.18 ***	251.24	778.99 **
		(253.19)	(1086.29)	(298.66)	(475.29)	(205.47)	(589.85)	(367.89)	(385.14)
	$\bar{ATT}$	1021.18	262.47	1150.77	1012.45	119.43	1790.94	234.92	795.91
Sample size	Quantity	895	347	1016	226	982	260	396	846
	Proportion	72%	28%	82%	18%	79%	21%	32%	68%

Note: Standard errors are shown in brackets; ***, **, and * are significant at the 1%, 5%, and 10% levels, respectively. $\bar{ATT}$ is the average value of ATT under different matching methods.

## Data Availability

The data presented in this study are available on request from the corresponding author.
